# 25-Hydroxyvitamin D Plasma Levels in Natural Populations of Pigmented and Partially Pigmented Land Iguanas from Galápagos (*Conolophus* spp.)

**DOI:** 10.1155/2022/7741397

**Published:** 2022-07-14

**Authors:** Cristina Di Giacomo, Leopoldo Pucillo, Christian Sevilla, Giorgio Fucci, Renato Massoud, Sergio Bernardini, Maurizio Fraziano, Gabriele Gentile

**Affiliations:** ^1^Clinical Biochemistry and Pharmacology Laboratory, National Institute for Infectious Diseases “L. Spallanzani”, Rome, Italy; ^2^Clinical Pathology Laboratory, A.O. San Camillo-Forlanini, Rome, Italy; ^3^Galápagos National Park Directorate, Puerto Ayora, Galápagos, Ecuador; ^4^Department of Experimental Medicine, University Tor Vergata, Rome, Italy; ^5^Department of Biology, University Tor Vergata, Rome, Italy

## Abstract

We report the first data on 25-hydroxyvitamin D plasma levels in natural populations of three species of land iguana endemic to the Galápagos Islands (*Conolophus marthae*, *C. subcristatus*, and *C. pallidus*). The pigment is present throughout the whole body in the skin of *C. subcristatus* and *C. pallidus*. On the contrary, pigment is not present in the skin of an extended part of the body in *C. marthae*. The only existing population of *C. marthae* is syntopic with a population of *C. subcristatus*, and the two species are closely related. These circumstances would suggest that, under the assumption that the species show a similar basking behavior and in the absence of compensatory mechanisms, lighter pigmentation should favor higher vitamin D levels. Thus, *C. marthae*, compared with *C. subcristatus* in Wolf Volcano, could show higher levels of 25(OH)D plasma levels, or equal, if compensatory mechanisms exist. The three species showed levels in the range of average values for healthy iguanas. However, contrary to the expectation, *C. marthae* consistently exhibited the lowest 25(OH)D plasma levels. We discuss possible factors affecting vitamin concentration and hypothesize that *C. marthae* may use the habitat to limit exposure to the high UVB irradiation at Wolf Volcano.

## 1. Introduction

The role of vitamin D is not limited to regulating calcium and bone health. Vitamin D may affect several systems [1] and may regulate antibacterial, antiviral, and anti-inflammatory innate immune responses [2, 3].

Vitamin D is not exclusive to animals, as it can be found in algae and some plants in the form of vitamin D_3_ (cholecalciferol) and, in minor amounts, as vitamin D_2_ (ergocalciferol), derived from contamination with fungi [4]. Vitamin D biosynthesis occurs along the sterol pathway in all organisms where the vitamin is present, with vitamin D_2_ originating from ergosterol exposed to ultraviolet B (UVB) radiation and vitamin D_3_ from 7-dehydrocholesterol exposed to UVB. In most animals, including humans, most of vitamin D (vitamin D_3_) is synthesized due to sunlight exposure, whereas dietary sources contribute to a less significant extent [5]. Once vitamin D_3_ is made in the skin or ingested from the diet, it undergoes further hydroxylations in the liver and kidney to form 25-hydroxyvitamin D (25(OH)D) and 1,25-dihydroxyvitamin D (1,25(OH)_2_D).

Skin pigmentation influences vitamin D_3_ levels because melanin competes for UVB photons with 7-dehydrocholesterol, which is converted into previtamin D_3_ and then vitamin D_3_ in the skin [6]. Many studies indicate that increased skin pigment can significantly reduce the ultraviolet ray-mediated synthesis of vitamin D_3_ in humans, also suggesting that lighter skin color evolved to optimize vitamin D_3_ production [6–10].

Once vitamin D_3_ is formed in the skin from previtamin D_3_, it is translocated from the skin into the circulation [11] in association with a vitamin D-specific binding globulin (DBP) [12]. If vitamin D_3_ in the skin is exposed to sunlight before its transfer into the circulation, it can be photodegraded. The most critical factor limiting the production of previtamin D_3_ in human skin is photochemical degradation of previtamin D_3_ rather than melanin pigmentation [13].

The vitamin D system in iguanian lizards was described by Laing and Fraser [14]. In iguanas, 25(OH)D is the major metabolite of vitamin D. It is the storage form of vitamin D. High concentration of vitamin D in the embryos and yolk of iguanas is possibly mediated by a mechanism similar to the one in birds [15] and indicates a role of the vitamin in embryogenesis [14]. Like mammals and birds, iguanas not exposed to ultraviolet light may suffer from vitamin D_3_ deficiency and consequent abnormalities in calcium metabolism, negatively affecting bone formation and growth of the embryo and the hatchability of eggs [16]. In general, low plasma vitamin D concentrations cause the metabolic bone disease, the main complex of diseases of reptiles in captivity [17].

Despite its importance, vitamin D in wild populations of iguanas has been poorly investigated. Filling this knowledge gap is particularly urgent as iguanas are a group of reptiles with a very high rate of endemism and a high percentage (61%) of species at risk of extinction [18]. Therefore, assessing vitamin D levels in wild populations of iguanas would prove highly beneficial for proper management, especially when it may imply *ex situ* phases, such as captive breeding and/or head start programs.

In the present work, we report the first data of 25(OH)D plasma levels in natural populations of three species of land iguanas endemic to Galápagos islands (*Conolophus marthae*, Cm; *C. subcristatus*, Cs; and *C. pallidus*, Cp). *Conolophus subcristatus* (Galápagos Land Iguanas) are distributed on the islands of Santa Cruz, Plaza Sur, Seymour Norte (introduced), Baltra (repatriated), Santiago (recently reintroduced), Isabela, and Fernandina. *Conolophus pallidus* (Barrington Land Iguanas) are limited to Santa Fe Island. *Conolophus marthae* (Galápagos Pink Land Iguanas), a recently described species [19, 20], is limited to the northern slopes of Wolf Volcano (1700 m, intersected by the Equator) on Isabela Island. *Conolophus marthae* lives in syntopy with a population of *C. subcristatus*, but the two species do not hybridize [21]. Species belonging to the genus *Conolophus* are herbivorous, with occasional integration of animal proteins by consuming insects or carrion [22]. Little is known about the diet of *C. marthae*, but preliminary data [23] indicate that the species is also herbivorous.

All three species are listed on the IUCN Red List. *Conolophus marthae* is listed as critically endangered [24], whereas *C. subcristatus* and *C. pallidus* are vulnerable [25, 26]. Although collected by opportunistic sampling, these data contribute further reference data for iguanas in the wild and are also of interest for management and conservation purposes for the Galápagos land iguana species. This is particularly true for *C. marthae*, for which a translocation to a new sanctuary area has been planned, following a few-years long head start program [27].

**Figure 1:**
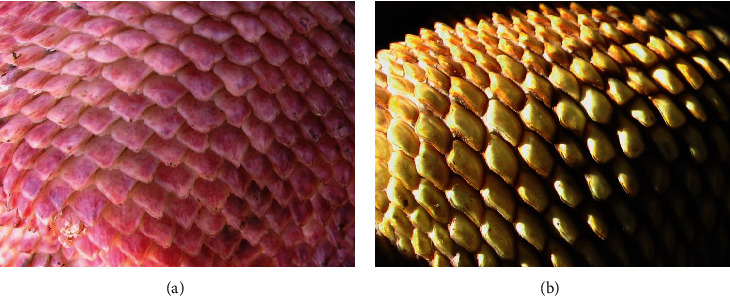
Skin pigmentation in Galápagos land iguanas *Conolophus marthae* (a) and *C. subcristatus* (b). Pink color in *C. marthae* is due to blood flowing in the deeper layers of scales.

Furthermore, the setting in Wolf Volcano offers an opportunity to comparatively discuss the results in the light of the different degrees of body pigmentation and other biological and ecological factors. Whereas the skin of *C. subcristatus* and *C. pallidus* is pigmented throughout the whole body, *C. marthae* is not (Figure 1) in most parts of the body, including the head, trunk, and legs [20]. These circumstances would suggest that, under the assumption that the species show a similar basking behavior and in the absence of compensatory mechanisms, light pigmentation should favor higher vitamin D levels. Thus, *C. marthae*, compared with *C. subcristatus* in Wolf Volcano, could show higher levels of 25(OH)D plasma levels, or equal, if compensatory mechanisms exist.

## 2. Materials and Methods

### 2.1. Ethics Statement

We performed animal manipulation and blood sampling according to a protocol that minimized animal stress, following the guidelines and with the approval of the Galápagos National Park Directorate. This Ecuadorian governmental authority administrates biodiversity in Galápagos. Samples were exported and imported under CITES export/import permits granted to Gabriele Gentile.

### 2.2. Sampling

**Figure 2:**
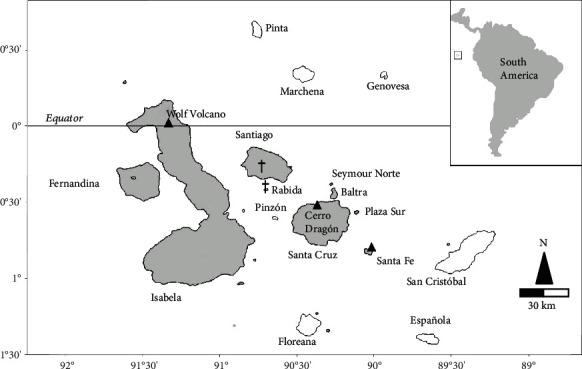
Galápagos archipelago. Islands where land iguanas occur or have occurred in historic times are in grey. Crosses indicate extinction. Black triangles indicate sampling locations.

We sampled iguanas opportunistically in three Galápagos Islands: Isabela (Wolf Volcano), Santa Cruz, and Santa Fe (Figure 2). Whereas the Galápagos Islands are grouped in the vicinity of the Equator, the sampling locations varied in altitude, which was approximately 1,700 m, 10 m, and 60 m for Isabela, Santa Cruz, and Santa Fe, respectively. This may determine some differences in the average UVB radiation between sites, with maximum UVB radiation occurring on the top of Wolf Volcano, up to 600 *μ*W/cm^2^, versus about 450 *μ*W/cm^2^ at the sea levels, measured at noon of a full sunny day (Solarmeter® Model 6.2 Sensitive UVB Meter). We sampled iguanas in different years: February 2005, 25 females and 25 males of *C. pallidus* at Santa Fe (SF) Island, and July 2005, 13 females and 21 males of *C. subcristatus* at Cerro Dragón (CD), Santa Cruz Island. We sampled *C. marthae* and *C. subcristatus* at the same time in Wolf Volcano: May 2009, 25 females and 24 males of *C. subcristatus*, 19 females and 31 males of *C. marthae* at Wolf Volcano (W), Isabela Island; July 2010, 5 females and 5 males of *C. subcristatus*, 4 females and 6 males of *C. marthae* at Wolf Volcano, Isabela Island; and June 2012, 25 females and 23 males of *C. subcristatus*, 21 females and 30 males of *C. marthae* at Wolf Volcano, Isabela Island. Although opportunistic, the sampling strategy maximized captures so that we could capture every sighted iguana. Additionally, a Passive Integrated Transponder (PIT) was implanted in every captured iguana. This permitted to exclude recaptured individuals from the subsequent statistical analyses. We determined gender by visual inspection of the cloaca for hemipenes' presence. We investigated females' reproductive status using a portable ultrasound machine (FUJIFILM SonoSite, Inc.), as in Gentile et al. [27]. We measured snout-vent length (SVL, cm) and weight (kg), for each individual and calculated a body condition index (BCI) as (body mass/SVL^3^) ×10^6^ [28]. As SVL is related to weight by an allometric relationship [29], we also estimated a scaled mass index of body condition (BCI_s_) as per Peig and coll. [30].

### 2.3. Blood Collection

We obtained blood samples from the caudal vein using heparinized syringes. We stored blood at +4°C for a few hours before centrifuging it to separate plasma. We kept plasma at -10°C while in the field for a few days and then at -80°C until we performed analyses of vitamin D_3_.

### 2.4. Total 25(OH)D Quantification

We measured total 25(OH)D levels at the National Institute of Infectious Diseases L. Spallanzani by using the chemiluminescence immunoassay (CLIA) Test Liaison® 25 OH Vitamin D Total (DiaSorin miniCD Liaison, Diasorin Inc., Minnesota, USA). The assay consists of an immunological direct competitive assay for the quantitative determination of total 25(OH)D in the serum or plasma with a 4 ng/ml detection limit. We carried out the analysis according to the protocol of the manufacturer.

### 2.5. Vitamin D_2_ and Vitamin D_3_ Levels' Assessment

We must consider some caveats when measuring total 25(OH)D. CLIA determination of total 25(OH)D assay does not discriminate between 25-hydroxyvitamin D_2_ and 25-hydroxyvitamin D_3_, nor can the assay discriminate between the vitamin D_3_ obtained by basking and from the diet. Indeed, in natural populations of herbivorous reptiles, UV exposure rather than diet plays the most critical role in determining vitamin D levels [17, 31]. Additionally, D_2_ supplementation is not as effective as D_3_ supplementation in raising plasma 25(OH)D levels in humans [32]. Nevertheless, to promote accuracy, we assessed plasmatic vitamin D_2_ and D_3_ levels in a random subsample of individuals of the three species (15 of *C. pallidus*, 9 of *C. marthae*, and 11 of *C. subcristatus* from Wolf Volcano). We used the HPLC (Spectra System, Thermo Separation Products, Waltham, MA, US) with UV/VIS detection, using a kit supplied by Eureka (Srl-Lab Division, Italy) following the protocol of the manufacturer. HPLC underestimates vitamin D concentration compared to CLIA determination [33]. Thus, we considered the absolute ratio D_3_/(D_2_+D_3_) as obtained from HPLC assay to estimate the proportion of vitamin D_3_ over the total D in the sample.

### 2.6. Statistical Analysis

After removing outliers, we applied the Shapiro-Wilk test to check for the normal distribution of 25(OH)D plasma concentration. Based on the Shapiro-Wilk test results (Table S1; Supplementary Materials), following an exploratory approach, we first performed a one-way analysis of variance (ANOVA) to test for significant differences between the means of 25(OH)D plasma levels of samples. For this purpose, regardless of sex, we treated each sample as a different coding (categorical) variable level. We then performed post hoc *t*-tests and applied the Bonferroni method that is type I error robust. Aware that some pairwise tests would be affected by low power due to a combination of effect and sample size, we also applied the less conservative Newman-Keuls test to reduce type II error and not to miss possible effects. Based on the analysis results (Table S2; Supplementary Information), we pooled *C. marthae* samples from 2009, 2010, and 2012. Similarly, we pooled *C. subcristatus* samples from Wolf Volcano collected in 2009, 2010, and 2012. We subtracted resampled individuals from the datasets. We then performed a two-way ANOVA to investigate the effect of species/population and sex as categorical variables on 25(OH)D plasma levels. We also conducted a two-way ANOVA to examine the effect of species/population and the presence of eggs as categorical variables on 25(OH)D plasma levels in females.

To evaluate intraindividual variability of 25(OH)D plasma level, we calculated the Pearson correlation coefficient (*r*). We performed a linear regression between individuals sampled in 2009 and recaptured in 2012 for *C. marthae* and *C. subcristatus* from Wolf Volcano.

We investigated the possible association between 25(OH)D plasma level and body size (SVL), as well as between 25(OH)D and BCI, by calculating the Pearson correlation coefficient (*r*). Because the three species show evident sexual dimorphism, with females being smaller than males, we calculated *r* coefficients separately for males and females.

Statistica ver. 8.0 (StatSoft, Inc.) and Past ver. 3.12 [34] packages were used for statistical analyses.

## 3. Results

The median values of the proportion of vitamin D_3_ over the total (D_2_+D_3_) estimated by HPLC in the three resampled groups ranged between 0.95 and 0.98, with D_2_ not detected in most cases. Thus, we reasonably concluded that 25(OH)D plasma concentration was primarily due to the D_3_ contribution. Plasma levels of 25(OH)D in different samples are reported in Table 1. The observed 25(OH)D values ranged from 17.3 to 357 ng/ml.

The one-way ANOVA indicated statistically significant differences between samples (*F*_Welch_ = 24.59; df. 66.96; *p* < <0.001).

**Figure 3:**
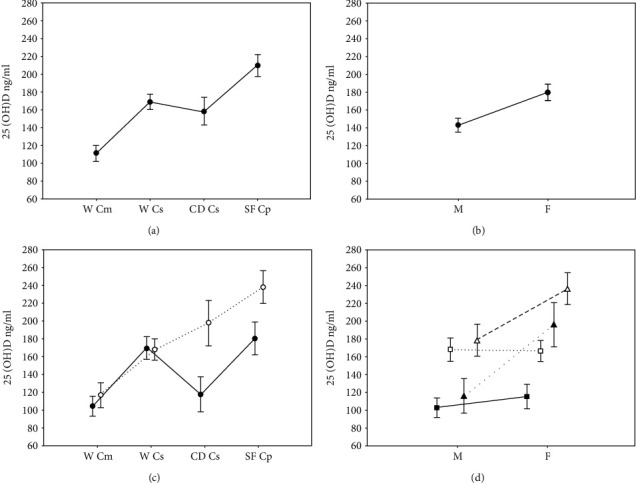
Two-way ANOVA to investigate the effect of species/populations and sex on 25(OH)D plasma level. Vertical bars denote 95% confidence intervals. (a) Main effect of species/population. (b) Main effect of sex. (c and d) Interactions between species/population and sex. In (c), open circles indicate females, and black circles indicate males. In (d) open triangles indicate *C. pallidus* (SF), black triangles indicate the CD population of *C. subcristatus*, open squares indicate the W *C. subcristatus* population, and black squares indicate *C. marthae*.

Results of the two-way ANOVA are in Figures 3(a)–3(d). A main effect was observed for species/population (*F* = 59.261; df. 3; *p* < <0.001). Post hoc tests indicated that the three species differed in their levels of 25(OH)D (*p* < <0.001), with *C. pallidus* showing the highest mean value and *C. marthae* the lowest. The 25(OH)D levels of the two populations of *C. subcristatus* (CD and W) were not different (post hoc *p* = 0.137). A main effect was also observed for sex (*F* = 37.922; df.1; *p* < <0.001), with females showing higher levels than males. A statistically significant interaction between species/population and sex was observed (*F* = 9.643; df. 3; *p* < <0.001). Females and males from Wolf Volcano did not differ in their 25(OH)D levels (post hoc *p* = 1.000), both in *C. marthae* and *C. subcristatus*. Females and males differed in the CD *C. subcristatus* population (post hoc *p* < <0.001) and in *C. pallidus* (post hoc *p* < <0.001).

No main effect for eggs (*F* = 1.209; df.1; *p* = 0.274) or a statistically significant interaction between species/population and eggs were observed (*F* = 0.002; df. 1; *p* = 0.967).

**Figure 4:**
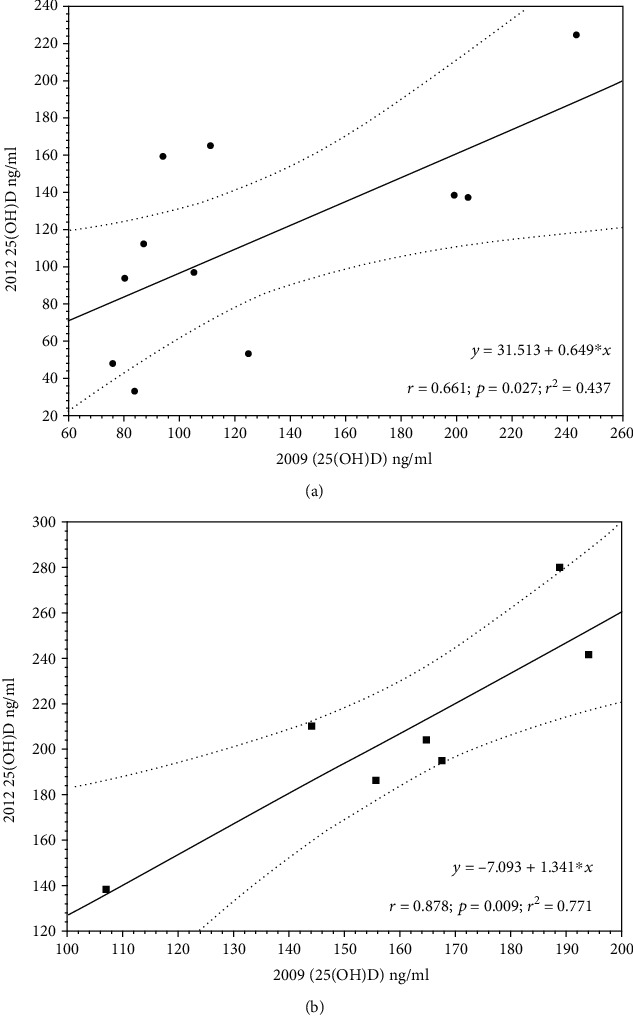
Linear regression between 25(OH)D concentrations in individuals captured in 2009 and recaptured in 2012 in Wolf Volcano. Dotted lines indicate 95% confidence interval. (a) *C. marthae* and (b) *C. subcristatus*.

The correlation coefficient and linear regression between 25(OH)D plasma levels in recaptured individuals for both *C. marthae* and *C. subcristatus* are reported in Figure 4.

Regardless of species/population and sex, 25(OH)D was not associated with SVL (*p* > >0.168 for all tests) with the only exception of *C. subcristatus* from Wolf Volcano (*r* = −0.316; *p* = 0.029). However, in this case, *r* became not statistically significant after a new threshold was established at *p* = 0.006 by the Bonferroni correction. Similarly, regardless of the method used, 25(OH)D level was not associated with BCI (*p* > >0.073 for all tests).

## 4. Discussion

Although captive management and clinical diagnosis would strongly benefit from studies of natural populations, little is published regarding mean plasma 25(OH)D levels in iguanas in the wild. From a comparison between species across literature, which requires caution given the diversity of assessment methods, the three *Conolophus* species showed average 25(OH)D plasma levels in the range of values reported for other iguanas. By combining values determined in wild and captive—but housed outside—individuals of five species of iguanids (*Pogona lesueurii*, *P*. *barbata*, *Chlamydosaurus kingii*, *Iguana iguana*, and *Cyclura cornuta*) Lang and Fraser [14] reported a mean plasma level of 25(OH)D equal to 105 nmol/l (corresponding to approximately 42 ng/ml). Higher values were observed in wild *I. iguana* in Costa Rica (approx. 146 ng/ml) [16] and in wild *Cyclura ricordii* and in wild and captive *C. cornuta cornuta* from Dominican Republic [36]. Mean concentrations were approximately 222 ng/ml (ranging between 100 and 448 ng/ml) for wild *C. ricordii*, 133 ng/ml (ranging between 104 and 148 ng/ml) for *C. c. cornuta*, and 127 ng/ml (ranging between 88 and 208 ng/ml) for captive *C. c. cornuta*. It seems reasonable that serum concentration of 25(OH)D—which may vary between and within iguana species—of at least 130 ng/ml could be considered normal for healthy iguanas [36].

We observed differences between 25(OH)D plasma levels of different *Conolophus* species. Considering the separate contribution of vitamin D_2_ and D_3_ to the total 25(OH)D plasma levels, we can conclude that vitamin D_2_ is much less abundant than vitamin D_3_ in the three species. Thus, most of the total amount of 25(OH)D estimated should be primarily due to vitamin D_3_ contribution, and differences observed between 25(OH)D levels should largely reflect differences in vitamin D_3_ levels. Even so, plasma levels of vitamin D_3_ may be determined by multiple factors. Without a specific experimental design, it is impossible to conclude that such differences are species-specific robustly. Indirect support to this hypothesis could be provided by the fact that, although the two populations of *C. subcristatus* were sampled at different times and locations, they showed average similar 25(OH)D plasma values.

Similarly, *C. marthae* samples consistently showed similarly low values, independently of the year of sampling. Further support could be provided by the remarkable differences between *C. marthae* and *C. subcristatus*, sampled syntopically and at the same time at Wolf Volcano, and by the strong correlation between 25(OH)D plasma levels in recaptured individuals found for both *C. marthae* and *C. subcristatus* (Figure 4). This evidence would suggest that such difference may have some genetic basis. Plasma levels of vitamin D_3_ are partly under genetic control; thus, they are partially inheritable (Wilson et al. 2011). Recently, evidence has been provided, showing that pigment genes may affect UVB-induced 25(OH)D concentrations [38].

However, for the opportunistic nature of sampling, we cannot provide an exhaustive discussion of factors that could explain the differences between samples collected from different species at different times. Perhaps we could invoke different sunlight and temperature conditions at sampling sites to explain such differences (Figure S1; Supplementary Materials), along with other, more species-dependent, physiological, ecological, and behavioral factors. For example, lizards can obtain detectable vitamin D_3_ from the diet [39], and Galápagos land iguanas differ in their diet across islands [22, 40]. We wonder if differences between the diets of the two syntopic species, *C. subcristatus* and *C. marthae* [23], may exist to explain the extreme differences between the 25(OH)D plasma levels of the two species. However, some reptiles seem capable of adjusting their exposure time to UVB irradiation depending on dietary intake of vitamin D_3_. It has been suggested that vitamin D_3_ synthesis may regulate basking behavior in turtles [41]. Evidence has been experimentally provided that the panther chameleon *Furcifer pardalis* may adjust basking behavior based on the vitamin D_3_ status [42]. Karsten et al. [43] suggested that this species can regulate the basking behavior by perceiving both UV radiation in the environment and their internal vitamin D_3_ status. Compensatory mechanisms have been described also for the skin of the shade-tolerant *Anolis lineotopus merope*, which seems to show a greater efficiency than that of the more heliophilic *A. sagrei* in UVB-induced vitamin D_3_ photobiosynthesis [39]. Such mechanisms allow the two species of *Anolis* to exhibit similar skin levels of vitamin D_3_. Given the strict conservation policy of the Galápagos National Park, an invasive experimental approach is impossible for *Conolophus*. Thus, we could not measure skin levels of vitamin D_3_. However, even if such mechanisms existed in *Conolophus*, it would remain to be explained why *C. marthae* and *C. subcristatus* would not show similar 25(OH)D plasma levels.

Although previous studies reported no sex- or species-dependent difference between mean values of plasma 25(OH)D levels [14], *Conolophus* species showed differences between sex, with females showing higher levels than males in two of the four populations investigated. We did not observe such a difference in the two populations from Wolf Volcano that belong to separate species. Interestingly, *C. marthae* and *C. subcristatus* in Wolf Volcano were sampled during their reproductive season [44]. In contrast, the CD population of *C. subcristatus* and *C. pallidus* were sampled when the species were far from reproduction [45]. The ANOVA analysis did not offer support for an increased vitamin D level in egg-carrying females. However, in both species, egg-carrying females seemed to show higher 25(OH)D mean values (data not shown). Thus, the lack of difference between males and females in Wolf populations might mirror an increase of vitamin D in males in response to reproduction. Cross-sectional studies in human and mammal animal models support the positive association between serum 25(OH)D level and sperm motility [35, 46]. Indeed, this possible association with reptiles deserves further attention.

25(OH)D levels were not associated with individual size, suggesting that size is not a limiting factor for the photobiosynthesis of vitamin D_3_, and if it is, this does not influence 25(OH)D plasma levels in these iguanas. Additionally, 25(OH)D levels were not associated with BCI, as expected in wild, healthy populations. A negative association between vitamin D and BCI was documented for humans and laboratory models when high BCI values are due to obesity, a pathological condition [47].

Contrary to the expectation, the partially pigmented *C. marthae* always showed levels lower than the fully pigmented *C. subcristatus* in Wolf Volcano. We consistently found such a difference over time. Excessive exposure to UVB can cause eye and skin damage, skin cancer, previtamin D_3_ photodegradation, and DNA damage [48]. We had never found evidence of eye and skin damage or skin cancer in *C. marthae* since 2005, when we started investigating this species [23]. However, we found a higher rate of DNA damage in *C. marthae*, likely in response to natural UVB irradiation as high as 500-600 *μ*W/cm^2^ [49]. Lower 25(OH)D plasma levels exhibited by *C. marthae* could reflect a higher rate of previtamin D_3_ photodegradation in this partially pigmented species and (or) a different usage of the habitat that would imply limited exposure to high UVB irradiation. If the extremely high UVB irradiation observed at Wolf Volcano can have detrimental effects, a partially pigmented species is expected to be more susceptible than a fully pigmented species, more protected from excessive exposure to UVB. Preliminary observations (Gentile, unpublished data) would indicate that *C. marthae* is more frequent in areas where vegetation coverage is thicker than for *C. subcristatus*. It is possible that *C. marthae* may prefer more vegetated areas also because here, iguanas might find a more shelter-enriched environment that could facilitate their basking-regulation behavior. Admittedly, the present correlative investigation does not allow drawing robust conclusions in this regard. This hypothesis awaits complete examination by a specific experimental design.

In conclusion, this work contributed important reference data on 25(OH)D plasma levels in wild populations of iguanas. It provided the first data on 25(OH)D plasma levels in the endangered *Conolophus* species. Despite the opportunistic collection of data and correlative nature of the analysis, this study provides indirect support to the possible species-specific variation in 25(OH)D plasma levels. It highlights a possible negative and positive association between 25(OH)D, pigmentation, and reproduction, delineating hypotheses for future investigations.

## Figures and Tables

**Table 1:** Plasma concentration of 25(OH)D in different samples. Plasma concentration is expressed in ng/ml.

	Year	*N*	Mean	Min	Max	St. dev.
*C. marthae* (Wolf Volcano)	2009	48	116.919	36.000	243.000	49.878
	2010	9	92.811	43.600	142.000	34.883
	2012	49	105.318	17.300	225.000	48.951

*C. subcristatus* (Wolf Volcano)	2009	48	161.554	57.600	238.700	36.411
	2010	9	131.678	84.200	211.000	40.289
	2012	46	183.900	90.000	245.500	39.087

*C. subcristatus* (Cerro Dragón)	2005	34	148.471	66.000	273.900	54.537
*C. pallidus* (Santa Fe)	2005	50	209.380	78.000	357.000	57.826

## Data Availability

Data are available upon request to the corresponding author Gabriele Gentile (gabriele.gentile@uniroma2.it).

## Supplementary Materials

Supplementary Materials In the Supplementary Material file, results of analyses, discussed but not shown in the main paper, are reported in the form of two tables (Tables S1 and S2) and one figure (Figure S1). Table S1: Shapiro-Wilk test to check for normal distribution of 25(OH)D plasma level. The first column reports population codes; the W statistics and the correspondent probability value are in the second and third columns, respectively. Table S2: one-way ANOVA post hoc tests. Above the diagonal: Neuman-Keuls *p* values. Below the diagonal: *p* values after *t*-tests with the Bonferroni correction. Probabilities statistically significant are in bold. W2009Cm indicates *C. marthae* (Cm) from Wolf Volcano (W) sampled in 2009. Similar rationale applies to other codes. Figure S1: 25(OH)D levels in *Conolophus marthae* (A), *C. subcristatus* and *C. pallidus* (B) from different samples (see caption of Table S2 for labels' explanation). Maximum temperature in Galápagos during sampling periods ((C) data from the Climatology Database of the Charles Darwin Foundation, https://www.darwinfoundation.org/en/datazone/climate). Dots indicate means, boxes indicate standard error, and vertical bars denote 2 × standard deviation.
