# Supplementary material for: 25-Hydroxyvitamin D Plasma Levels in Natural Populations of Pigmented and Partially Pigmented Land Iguanas from Galápagos (Conolophus spp.)
Source: Biomed Res Int. 2022 Jul 14;2022:7741397. doi: 10.1155/2022/7741397 (PMC9303120; doi:10.1155/2022/7741397)
Supplement: Supplementary Materials — In the Supplementary Material file, results of analyses, discussed but not shown in the main paper, are reported in the form of two tables (Tables S1 and S2) and one figure (Figure S1). Table S1: Shapiro-Wilk test to check for normal distribution of 25(OH)D plasma level. The first column reports population codes; the W statistics and the correspondent probability value are in the second and third columns, respectively. Table S2: one-way ANOVA post hoc tests. Above the diagonal: Neuman-Keuls p values. Below the diagonal: p values after t-tests with the Bonferroni correction. Probabilities statistically significant are in bold. W2009Cm indicates C. marthae (Cm) from Wolf Volcano (W) sampled in 2009. Similar rationale applies to other codes. Figure S1: 25(OH)D levels in Conolophus marthae (A), C. subcristatus and C. pallidus (B) from different samples (see caption of Table S2 for labels' explanation). Maximum temperature in Galápagos during sampling periods ((C) data from the Climatology Database of the Charles Darwin Foundation, https://www.darwinfoundation.org/en/datazone/climate). Dots indicate means, boxes indicate standard error, and vertical bars denote 2 × standard deviation. [file 7741397.f1.docx]

**25-hydroxyvitamin D plasma levels in natural populations of** **pigmented and partially pigmented land iguanas from Galápagos (*Conolophu****s* **spp.)**

Cristina Di Giacomo^1,2^, Leopoldo Pucillo^1,2^, Christian Sevilla^3^, Giorgio Fucci^4^, Renato Massoud^4^, Sergio Bernardini^4^, Maurizio Fraziano^5^, and Gabriele Gentile^5 *^

*^1^ Clinical Biochemistry and Pharmacology Laboratory, National Institute for Infectious Diseases "L. Spallanzani", Rome, Italy*

*^2^ Clinical Pathology Laboratory, A.O. San Camillo-Forlanini, Rome, Italy,*

*^3^ Galápagos National Park Directorate, Puerto Ayora, Galápagos, Ecuador*

*^4^ Department of Experimental Medicine, University Tor Vergata, Rome, Italy*

*^5^ Department of Biology, University Tor Vergata, Rome, Italy*

** Corresponding author:* [***gabriele.gentile@uniroma2.it***](mailto:gabriele.gentile@uniroma2.it)***)***

[**ORCID: 0000-0002-1045-6816**](https://orcid.org/0000-0002-1045-6816)

**SUPPLEMENTARY MATERIAL**

|  | W | *p* |
| --- | --- | --- |
| W 2009 Cm | 0.954 | 0.056 |
| W 2010 Cm | 0.936 | 0.536 |
| W 2012 Cm | 0.984 | 0.725 |
| W 2009 Cs | 0.989 | 0.921 |
| W 2010 Cs | 0.943 | 0.613 |
| W 2012 Cs | 0.951 | 0.052 |
| CD 2005 Cs | 0.963 | 0.307 |
| SF 2005 Cp | 0.993 | 0.987 |

**Table S1.** Shapiro-Wilk test to check for normal distribution of 25(OH)D plasma concentration. The first column reports population codes; the W statistics and the correspondent probability value are in the second and third columns, respectively.

**Table S2.** One-way ANOVA post-hoc tests. Above the diagonal: Neuman-Keuls *p*-values. Below the diagonal: *p*-values after *t*-tests with Bonferroni correction. Probabilities statistically significant are in bold. W2009Cm indicates *C. marthae* (Cm) from Wolf Volcano (W) sampled in 2009. Similar rationale applies to other codes.

|  | W 2009 Cm | W 2010 Cm | W 2012 Cm | W 2009 Cs | W 2010 Cs | W 2012 Cs | CD 2005 Cs | SF 2005 Cp |
| --- | --- | --- | --- | --- | --- | --- | --- | --- |
| W 2009 Cm | - | 0.207 | 0.414 | **0.009** | 0.299 | **<<0.001** | 0.068 | **<<0.001** |
| W 2010 Cm | 1.000 | - | 0.379 | **<<0.001** | **0.032** | **<<0.001** | **0.001** | **<<0.001** |
| W 2012 Cm | 1.000 | 1.000 | - | **0.001** | 0.152 | **<<0.001** | **0.013** | **<<0.001** |
| W 2009 Cs | **<<0.001** | **0.002** | **<<0.001** | - | 0.089 | 0.116 | 0.357 | **0.002** |
| W 2010 Cs | 1.000 | 1.000 | 1.000 | 1.000 | - | **0.001** | 0.237 | **<<0.001** |
| W 2012 Cs | **<<0.001** | **<<0.001** | **<<0.001** | 0.668 | 0.081 | - | **0.034** | 0.073 |
| CD 2005 Cs | 0.096 | 0.057 | **0.002** | 1.000 | 1.000 | **0.032** | - | **<<0.001** |
| SF 2005 Cp | **<<0.001** | **<<0.001** | **<<0.001** | **<<0.001** | **<<0.001** | 0.262 | **<<0.001** | - |
|  |  |  |  |  |  |  |  |  |

**
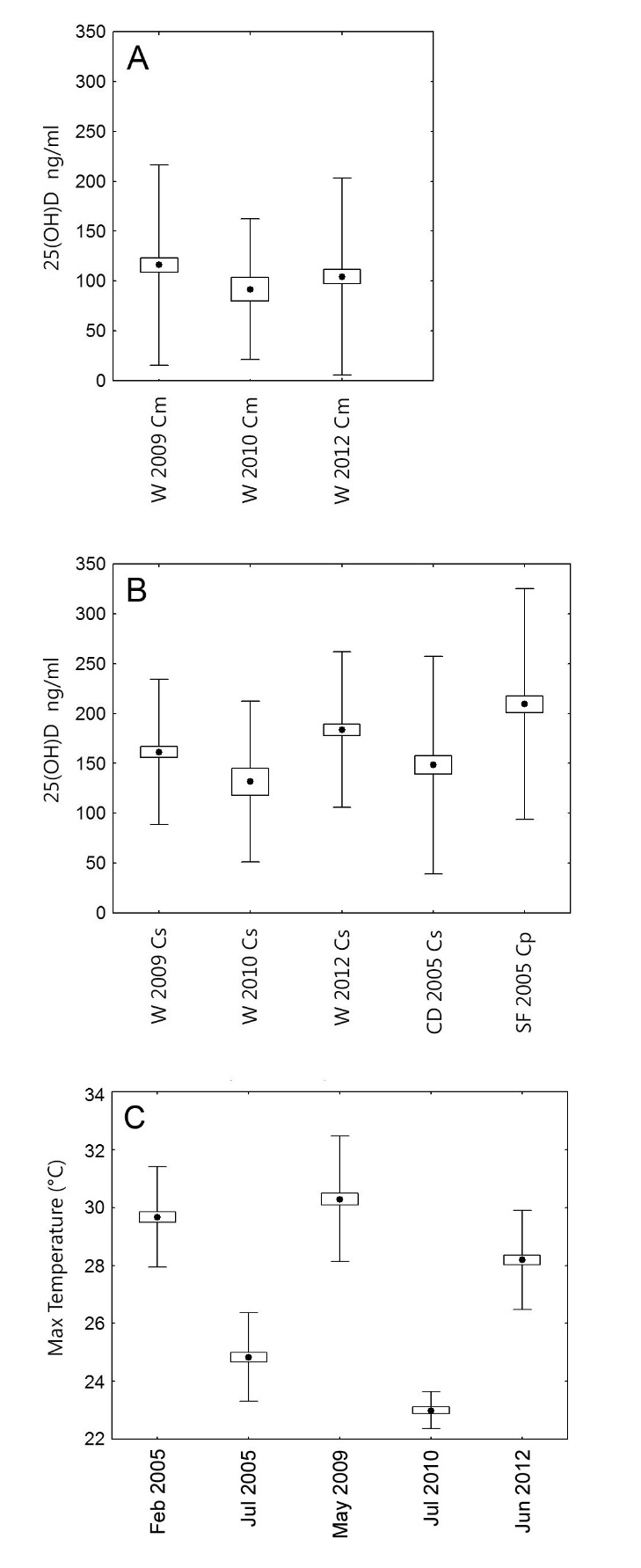
**

**Figure S1**. 25(OH)D levels in *Conolophus marthae* (A), *C. subcristatus* and *C. pallidus* (B) from different samples (see caption of Table S2 for labels’ explanation). Maximum Temperature in Galápagos during sampling periods (C, data from the Climatology Database of the Charles Darwin Foundation, <https://www.darwinfoundation.org/en/datazone/climate>). Dots indicate means, boxes indicate standard error, and vertical bars denote 2 x standard deviation.
